# Association of nurse-led targeted sedation-analgesia management with ventilation duration and sedation quality in mechanically ventilated patients

**DOI:** 10.1371/journal.pone.0353344

**Published:** 2026-07-10

**Authors:** Xin Li, Huiyi Zhang, Hongyan Zhang, Ruxin Jiang, Su Wu, Shaoru Chen, Hui Zhi

**Affiliations:** 1 Department of Anesthesiology, Henan Provincial People’s Hospital, Zhengzhou, China; 2 Department of Ophthalmology, Henan Provincial People’s Hospital, Zhengzhou, China; University of Sao Paulo: Universidade de Sao Paulo, BRAZIL

## Abstract

**Purpose:**

Variability in sedation–analgesia assessment and titration is common in mechanically ventilated ICU patients and may contribute to unnecessary deep sedation and delayed ventilator liberation. This study examined the association between a nurse-led targeted sedation-analgesia management workflow and clinical outcomes in an Intensive Care Unit (ICU).

**Methods:**

This single-center retrospective observational cohort study was conducted and reported in accordance with the STROBE statement. Adult patients receiving invasive mechanical ventilation for ≥24 hours in the ICU from January 1, 2024 to December 31, 2025 were included. Patients were classified into a usual-care group (physician-directed care) or a nurse-led targeted management group using prespecified documentation-based operational criteria, including daily RASS targets, assessment frequency, nurse-driven titration, and closed-loop reassessment. Primary outcomes were duration of invasive mechanical ventilation and sedation target attainment (proportion of RASS assessments within target). Secondary outcomes included deep sedation exposure (RASS ≤ −3), agitation exposure (RASS ≥ +1), ICU length of stay, delirium, unplanned extubation, reintubation within 48 hours, tracheostomy, and 28-day mortality.

**Results:**

A total of 102 patients were analyzed (usual-care group, n = 48; nurse-led group, n = 54). The nurse-led group had shorter mechanical ventilation duration (4.3 [3.2–6.3] vs 5.9 [4.3–8.7] days; P < 0.01) and higher sedation target attainment (74.0% ± 11.5% vs 61.5% ± 12.8%; P < 0.01). Deep sedation exposure and agitation exposure were lower in the nurse-led group (16.1% ± 9.1% vs 27.6% ± 10.9%; P < 0.01, and 9.1% ± 6.0% vs 12.4% ± 7.3%; P = 0.03, respectively), and ICU length of stay was shorter (8.0 ± 4.2 vs 9.7 ± 4.9 days; P = 0.02). Delirium was numerically lower but not statistically significant (22.22% vs 35.42%; P = 0.19), and no significant differences were observed in unplanned extubation, reintubation within 48 hours, tracheostomy, or 28-day mortality. After adjustment, nurse-led targeted management was associated with shorter ventilation duration (adjusted ratio, 0.79; 95% CI, 0.67–0.93; P = 0.004), whereas the association with delirium was not significant (adjusted OR, 0.58; 95% CI, 0.26–1.30; P = 0.190).

**Conclusion:**

In this retrospective cohort study, nurse-led targeted sedation-analgesia management was associated with higher sedation target attainment, lower exposure to deep sedation and agitation, shorter invasive mechanical ventilation duration, and shorter ICU stay. No increase in measured safety events was detected. Given the retrospective design, small sample size, and limited adjustment, these findings should be interpreted as associations rather than evidence of causal effectiveness.

## 1. Introduction

Invasive mechanical ventilation is a cornerstone of life support in intensive care units, and most ventilated patients require analgesia and sedation to relieve pain and anxiety, facilitate ventilator synchrony, and ensure safety [1]. Contemporary guidelines emphasize an assessment-driven approach featuring analgesia-first strategies, goal-directed sedation, avoidance of unnecessary deep sedation, and continuous monitoring to optimize ventilation-related outcomes. Objective tools are fundamental to this approach. The Richmond Agitation–Sedation Scale (RASS) has demonstrated strong reliability and validity for quantifying sedation depth in both ventilated and non-ventilated adult ICU patients [1,2].Pain is a major driver of sedative requirements; behavioral pain tools such as the Critical-Care Pain Observation Tool (CPOT) show robust measurement properties in critically ill patients unable to self-report pain, supporting more precise analgesic titration [3,4].

Deep sedation and acute brain dysfunction, particularly delirium, are closely associated with adverse outcomes. Delirium has been shown to independently predict mortality and prolonged hospitalization among mechanically ventilated ICU patients, underscoring the need to integrate brain dysfunction prevention and management into analgesia–sedation practice [5]. Meanwhile, strengthening protocolized sedation and liberation workflows can improve ventilation-related outcomes. Daily interruption of sedative infusions has been associated with shorter ventilation duration and reduced ICU length of stay [6]. Pairing spontaneous awakening trials with spontaneous breathing trials further improved outcomes in a randomized trial of mechanically ventilated patients, highlighting the value of integrating goal-directed sedation with ventilator weaning processes [7]. Regarding sedative selection, compared with benzodiazepines, dexmedetomidine has been linked to less acute brain dysfunction and improved goal attainment in randomized controlled settings [8].

Despite these advances, substantial practice variation remains in real-world critical care settings, including in the ICU, particularly in assessment frequency, timeliness of titration, and execution of closed-loop reassessment.Earlier PAD guidance also advocated integrated, patient-centered, protocol-based management to reduce practice variability [9].In addition, analgesia-based, goal-directed protocols led by nurses have been shown to be feasible and safe and may reduce deep-sedation exposure.Therefore, this study aimed to evaluate the effectiveness of a nurse led targeted sedation–analgesia management program in mechanically ventilated patients in the ICU, focusing on sedation quality, ventilation duration, and safety outcomes [10].Therefore, this study aimed to examine the association between a nurse-led targeted sedation-analgesia management workflow and ventilation duration, sedation quality, and safety outcomes in mechanically ventilated ICU patients.

## 2. Methods

### 2.1. Study design and setting

This was a single-center retrospective cohort study conducted in the Intensive Care Unit (ICU) at our hospital over a 2-year period (January 1, 2024 to December 31, 2025). Patients were retrospectively categorized as receiving usual care (usual-care group) or nurse-led targeted sedation-analgesia management (nurse-led group) based on prespecified operational criteria applied to electronic health records and nursing documentation. This study was approved by the Ethics Committee of Henan Provincial People’s Hospital. Given the retrospective design and de-identified data processing, informed consent was waived.The manuscript was prepared in accordance with the Strengthening the Reporting of Observational Studies in Epidemiology (STROBE) statement.

### 2.2. Participants

#### 2.2.1. Inclusion criteria.

Patients were included if they met all of the following criteria:

(1) Age ≥ 18 years; (2) Invasive mechanical ventilation for ≥ 24 hours; (3) Analgesia and/or sedation initiated within 24 hours of ICU admission; (4) Nursing documentation available for sedation assessment (RASS) and pain assessment (NRS/CPOT/BPS), sufficient for exposure classification and outcome ascertainment.

#### 2.2.2. Exclusion criteria.

Patients were excluded if they met any of the following criteria:

(1) Mandatory indications for Exposure (e.g., refractory intracranial hypertension,or status epilepticus, prolonged neuromuscular blockade); (2) Brain death or persistent coma at ICU admission; (3) Primary alcohol/drug withdrawal requiring a specialized sedation protocol; (4) Invasive mechanical ventilation < 24 hours or death within 24 hours of ICU admission; (5) pregnancy; and (6) missing key sedation/pain documentation precluding exposure classification or outcome assessment.

### 2.3. Exposure and group definitions

Control group (usual care): Sedation–analgesia targets and infusion adjustments were primarily physician-directed. Nurses documented routine assessments and administered medications as ordered, without a standardized nurse-driven titration algorithm.

Nurse-led group (nurse-led targeted management): The program followed an analgesia-first, goal-directed framework. Physicians specified a daily target sedation range (typically RASS −2–0, adjustable per clinical condition). Nurses performed structured pain, sedation, and delirium assessments and titrated sedatives/analgesics within physician-defined targets using a standardized algorithm, followed by timely reassessment (closed-loop workflow). Prespecified safety triggers required physician notification (e.g., persistent severe agitation despite titration, hemodynamic instability, or airway safety concerns).

Operational criteria: To retrospectively ascertain likely exposure to a nurse-led targeted workflow, patients were classified as receiving nurse-led targeted management if they met ≥ 3 of the following criteria within a 24-hour window during invasive ventilation: (1) a documented daily RASS target in orders/progress notes; (2) RASS assessments ≥6 per 24 hours and pain assessments ≥4 per 24 hours; (3) ≥2 documented sedative/analgesic infusion-rate titrations per 24 hours; and (4) a documented reassessment within 2 hours after titration. Patients not meeting ≥3 criteria were assigned to the usual-care group. These documentation-based indicators were used for exposure classification only and were not interpreted as independent evidence of clinical benefit.

### 2.4. Data collection

Data were extracted from electronic health records, medication administration records, ventilator logs, and nursing documentation systems. Collected variables included age, sex, APACHE II and SOFA scores at ICU admission, admission category (medical vs surgical), major diagnoses (including sepsis/septic shock and ARDS), vasopressor use within 24 hours, chronic lung disease, and sedative class exposure (propofol, dexmedetomidine, benzodiazepines). Documentation-based process indicators were also extracted from nursing timestamps (assessment counts, titration counts, and reassessment within 2 hours after titration) and are presented descriptively because they overlap with the retrospective exposure ascertainment criteria.

### 2.5. Outcomes

Primary outcomes were (1) duration of invasive ventilation (days), and (2) documentation-derived sedation target attainment (%), defined as the proportion of RASS assessments within the physician-defined daily target range.

Secondary outcomes include Exposure (percentage of RASS assessments ≤ −3), exposure to agitation (percentage of RASS assessments ≥ +1), ICU length of stay (days), delirium incidence (any positive delirium assessment), unplanned extubation, reintubation within 48 hours, tracheostomy, and 28-day mortality. Medication exposure outcomes included sedative exposure days and opioid exposure days.

### 2.6. Statistical analysis

Continuous variables are presented as mean ± standard deviation (SD) or median (Interquartile range[IQR]), as appropriate. Between-group comparisons used the Student t test or Mann-Whitney U test for continuous variables and χ² or two-sided Fisher exact test for categorical variables (Fisher exact test was reported for categorical outcomes). A generalized linear model (Gamma distribution with log link) was used to evaluate the association between nurse-led management and ventilation duration, reporting adjusted ratios with 95% confidence intervals (CIs); this model included nurse-led management, APACHE II (per 5-point increase), vasopressor use within 24 hours, and ARDS. Multivariable logistic regression was used for delirium, reporting adjusted odds ratios (ORs) with 95% CIs; this model included nurse-led management, APACHE II (per 5-point increase), age (per 10-year increase), and benzodiazepine exposure. Covariate selection was intentionally parsimonious because the total sample size and number of outcome events were limited. Exploratory Kaplan-Meier curves were generated for time to successful extubation and compared using the log-rank test; because competing events were not formally modeled, these curves were interpreted cautiously and not used for primary inference. Two-sided P < 0.05 was considered statistically significant. Statistical analyses were performed using R software (version 4.3.2; R Foundation for Statistical Computing, Vienna, Austria).

## 3. Results

### 3.1. Patient selection and study population

**Fig 1:**
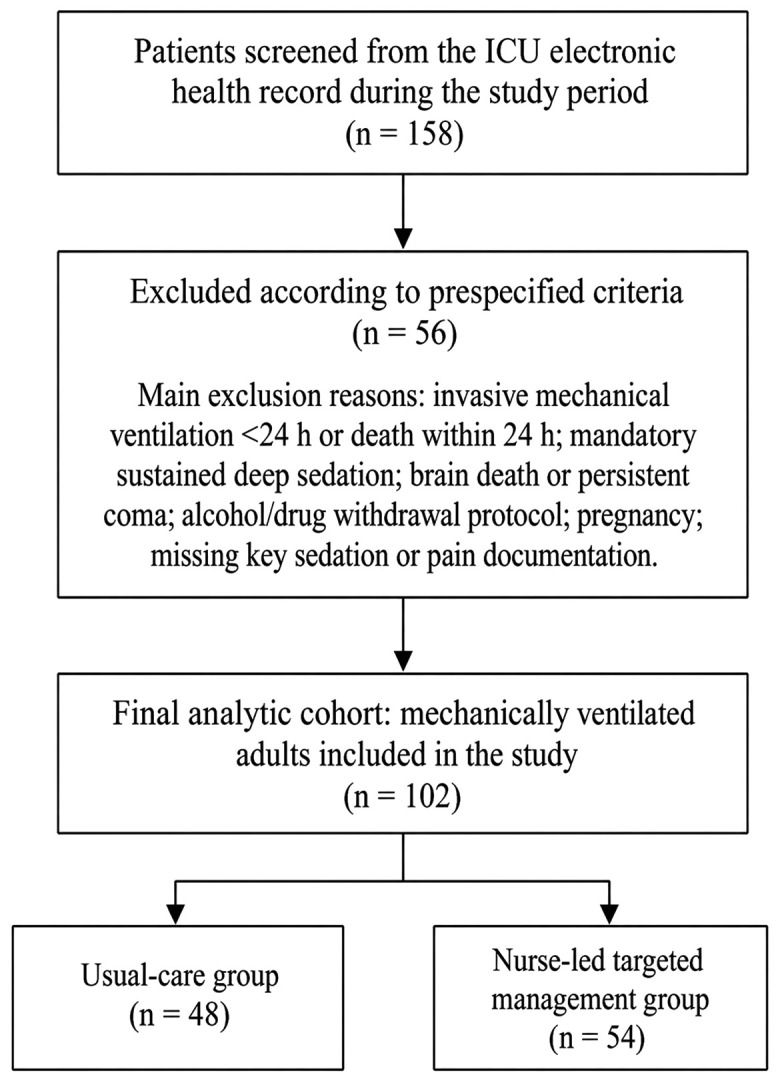
Patient selection flow diagram reported according to the STROBE framework. Because the retrospective screening log was unavailable, total screened and reason-specific exclusion counts are reported as not available rather than imputed.

The patient selection process is summarized in Fig 1. A total of 102 adult patients receiving invasive mechanical ventilation in the ICU were included in the final analysis. According to the predefined documentation-based operational criteria used for retrospective exposure ascertainment, 48 patients were categorized as the usual-care group and 54 as the nurse-led targeted management group. The retrospective screening log did not record the total number of potentially eligible admissions or reason-specific exclusion counts; therefore, these values are reported as not available rather than imputed.

### 3.2. Baseline characteristics

No statistically significant between-group differences were observed in measured baseline variables, including demographics, illness severity (APACHE II and SOFA), admission category (medical vs surgical), major diagnoses (sepsis/septic shock and ARDS), vasopressor use within 24 hours, chronic lung disease, and primary sedative class exposure (all P > 0.05) (Table 1). However, as in any nonrandomized retrospective study, residual clinical imbalance cannot be excluded.

**Table 1:** Baseline characteristics.

Variable	Usual-care (n = 48)	Nurse-led (n = 54)	P value
Age	60.90 ± 15.60	60.10 ± 14.7	0.79
Male	30 (62.50%)	32 (59.26%)	0.74
APACHE II	19.10 ± 6.20	18.30 ± 6	0.51
SOFA	7 (5–9)	7 (5–8)	0.58
Medical admission	27 (56.25%)	30 (55.56%)	0.94
Surgical admission	21 (43.75%)	24 (44.44%)	0.94
Sepsis/septic shock	16 (33.33%)	17 (31.48%)	0.84
ARDS	9 (18.75%)	10 (18.52%)	0.98
Vasopressor use within 24 h	23 (47.92%)	24 (44.44%)	0.72
Chronic lung disease	8 (16.67%)	7 (12.96%)	0.59
Primary sedative class exposure			0.62
Propofol	26 (54.17%)	31 (57.41%)	
Dexmedetomidine	14 (29.17%)	17 (31.48%)	
Benzodiazepines	8 (16.67%)	6 (11.11%)	

### 3.3. Documentation-based process indicators

**Fig 2:**
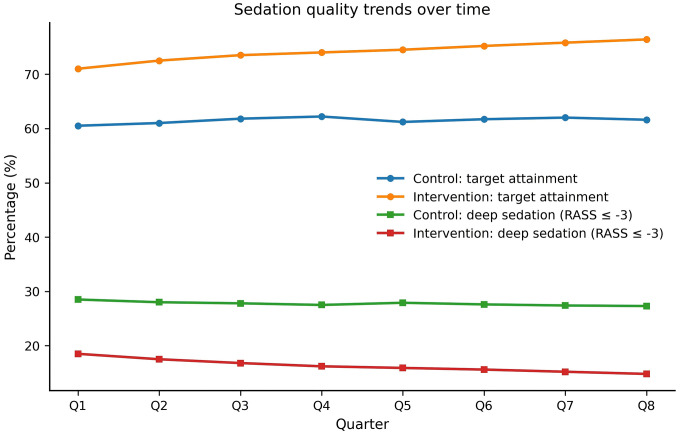
Sedation quality trends over time. Quarterly trends in sedation target attainment (%**) and deep sedation exposure (RASS ≤ −3, %) in the usual-care and nurse-led groups.** Target attainment was defined as the proportion of RASS assessments within the physician-defined daily target range.

Documentation-based workflow indicators differed between groups by design, because these indicators contributed to retrospective exposure classification. RASS assessments per 24 hours were more frequent in the nurse-led group (8 [7 –10] vs 5 [4 –6]; P < 0.01), as were pain assessments per 24 hours (5 [4 –6] vs 3 [2 –4]; P < 0.01) and delirium assessments per day (2 [1 –2] vs 1 [0–1]; P < 0.01). During the first 72 hours of invasive ventilation, sedative/analgesic infusion titrations occurred more often (6 [4 –8] vs 3 [2 –5]; P < 0.01), and reassessment within 2 hours after titration was more common (73.5% ± 15.1% vs 41.2% ± 18.6%; P < 0.01). Daily RASS targets were documented more frequently in orders/progress notes (92.59% vs 45.83%; P < 0.01) (Table 2). These process indicators are presented descriptively and should not be interpreted as independent evidence of intervention efficacy. Consistent with these process indicators, quarterly trends showed higher target attainment and lower deep-sedation exposure in the nurse-led group over time (Fig 2).

**Table 2:** Documentation-based workflow indicators used for exposure ascertainment and early descriptive characterization.

Measure	Usual-care (n = 48)	Nurse-led (n = 54)	P value
RASS assessments per 24 h	5 (4–6)	8 (7–10)	<0.01
Pain assessments per 24 h	3 (2–4)	5 (4–6)	<0.01
Delirium assessments per day	1 (0–1)	2 (1–2)	<0.01
Sedation/analgesia infusion titrations in first 72 h	3 (2–5)	6 (4–8)	<0.01
Reassessment within 2 h after titration, patient-level %	41.20 ± 18.60	73.50 ± 15.10	<0.01
Daily RASS target documented	22 (45.83%)	50 (92.59%)	<0.01

### 3.4. Primary outcomes

**Fig 3:**
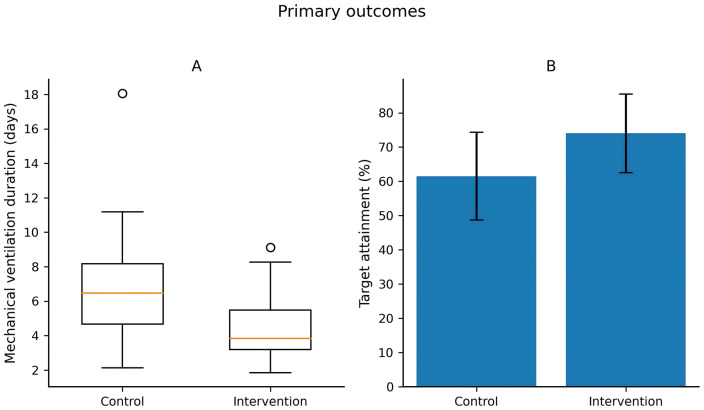
Primary outcomes. (A) Mechanical ventilation duration (days) presented as boxplots for the usual-care and nurse-led groups. (B) Documentation-derived sedation target attainment (%) presented as mean ± SD.

Mechanical ventilation duration was shorter in the nurse-led group than in the usual-care group (4.3 [3.2–6.3] vs 5.9 [4.3–8.7] days; P < 0.01). Documentation-derived sedation target attainment was higher in the nurse-led group (74.0% ± 11.5% vs 61.5% ± 12.8%; P < 0.01) (Table 3, Fig 3).

**Table 3:** Clinical outcomes.

Outcome	Usual-care (n = 48)	Nurse-led(n = 54)	P value
Mechanical ventilation duration, days	5.9 (4.3–8.7)	4.3 (3.2–6.3)	<0.01
Documentation-derived sedation target attainment	61.5 ± 12.8	74.0 ± 11.5	<0.01
Deep sedation exposure (RASS ≤ −3)	27.6 ± 10.9	16.1 ± 9.1	<0.01
Agitation exposure (RASS ≥ +1)	12.4 ± 7.3	9.1 ± 6.0	0.03
ICU length of stay, days	9.7 ± 4.9	8.0 ± 4.2	0.02
Delirium (any positive assessment)	17 (35.42%)	12 (22.22%)	0.19
Unplanned extubation	3 (6.25%)	2 (3.70%)	0.66
Reintubation within 48 h	5 (10.42%)	4 (7.41%)	0.73
Tracheostomy	8 (16.67%)	6 (11.11%)	0.57
28-day mortality	8 (16.67%)	6 (11.11%)	0.57

### 3.5. Secondary outcomes and safety outcomes

Exposure to deep sedation (RASS ≤ −3) was lower in the nurse-led group (16.1% ± 9.1% vs 27.6% ± 10.9%; P < 0.01), and exposure to agitation (RASS ≥ +1) was also lower (9.1% ± 6.0% vs 12.4% ± 7.3%; P = 0.03). ICU length of stay was shorter in the nurse-led group (8.0 ± 4.2 vs 9.7 ± 4.9 days; P = 0.02) (Table 3). Delirium occurred less frequently in the nurse-led group (22.22% vs 35.42%), but the difference was not statistically significant (P = 0.19). Given that delirium assessments were performed more frequently in the nurse-led group, this finding should be interpreted cautiously because differential ascertainment is possible. No significant between-group differences were observed in unplanned extubation (3.70% vs 6.25%; P = 0.66), reintubation within 48 hours (7.41% vs 10.42%; P = 0.73), tracheostomy (11.11% vs 16.67%; P = 0.57), or 28-day mortality (11.11% vs 16.67%; P = 0.57) (Table 3).

### 3.6. Medication exposure and multivariable analyses

**Fig 4:**
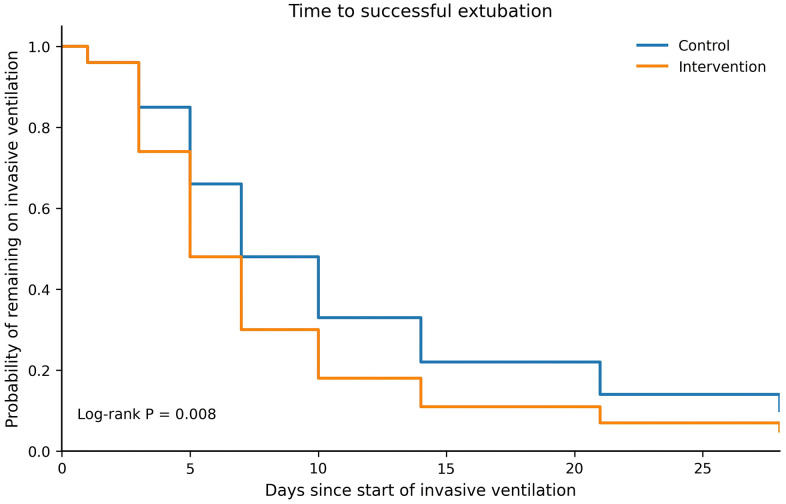
Exploratory time to successful extubation. Exploratory Kaplan-Meier curves showing the probability of remaining on invasive mechanical ventilation over time in the usual-care and nurse-led groups. Successful extubation was defined as extubation without reintubation within 48 hours. Because death and tracheostomy were not modeled as competing events, the log-rank P value shown on the plot should be interpreted cautiously.

Sedative exposure days were fewer in the nurse-led group (3.9 [2.9–5.7] vs 5.2 [4.0–7.6] days; P = 0.01), whereas opioid exposure days were similar between groups (5.7 [4.1–7.8] vs 6.3 [4.6–8.5] days; P = 0.20). Because medication exposure duration is partly conditioned on overall ventilation duration and ICU trajectory, these findings should be interpreted as contextual rather than independent evidence of benefit. Benzodiazepine exposure was numerically lower without statistical significance (11.11% vs 16.67%; P = 0.41) (Table 4). After adjustment in a Gamma generalized linear model (log link), nurse-led targeted management was associated with shorter ventilation duration (adjusted ratio, 0.79; 95% CI, 0.67–0.93; P = 0.004) (Table 5). Exploratory Kaplan-Meier analysis suggested earlier successful extubation in the nurse-led group (log-rank P = 0.008), but this result should be interpreted cautiously because competing events such as death and tracheostomy were not formally modeled (Fig 4). In the adjusted logistic regression model for delirium, nurse-led management showed a nonsignificant trend toward reduced delirium (adjusted OR, 0.58; 95% CI, 0.26–1.30; P = 0.190) (Table 5). Different covariate sets were prespecified for the two multivariable models according to outcome-specific clinical relevance and event-per-variable constraints.

**Table 4:** Medication exposure.

Measure	Usual-care (n = 48)	Nurse-led (n = 54)	P value
Sedative exposure days	5.20 (4.00–7.60)	3.90 (2.90–5.70)	0.01
Opioid exposure days	6.30 (4.60–8.50)	5.70 (4.10–7.80)	0.20
Dexmedetomidine use	14 (29.17%)	17 (31.48%)	0.80
Benzodiazepine exposure	8 (16.67%)	6 (11.11%)	0.41
Propofol use	26 (54.17%)	31 (57.41%)	0.74

**Table 5:** Multivariable regression analyses.

Outcome	Effect estimate	95% CI	P value
Mechanical ventilation duration			
Nurse-led targeted management	0.79	0.67–0.93	0.004
APACHE II (per 5-point increase)	1.12	1.02–1.24	0.021
Vasopressor use	1.18	1.01–1.38	0.034
ARDS	1.15	0.97–1.36	0.110
Delirium			
Nurse-led targeted management	0.58	0.26–1.30	0.190
APACHE II (per 5-point increase)	1.24	1.01–1.53	0.042
Age (per 10-year increase)	1.16	0.92–1.47	0.210
Benzodiazepine exposure	1.63	0.66–4.05	0.290

## 4. Discussion

In this retrospective observational cohort study of mechanically ventilated patients in the AICU, nurse-led targeted sedation-analgesia management was associated with shorter duration of mechanical ventilation, higher sedation target attainment, and lower exposure to deep sedation and agitation, while no significant differences were observed in key safety outcomes such as unplanned extubation, reintubation, tracheostomy, or 28-day mortality. These findings align with the direction of the ICU Liberation Collaborative, which emphasizes process-driven improvements in analgesia-sedation and ventilator liberation to reduce oversedation and improve patient outcomes. Evidence from large-scale implementation suggests that higher-quality bundle delivery is associated with improved outcomes, including reduced ventilator use, coma, and delirium [11]. Our results are also consistent with evidence from systematic reviews and meta-analyses indicating that protocolized sedation can shorten ventilation duration and improve selected clinical outcomes [12]. More recent syntheses similarly suggest that protocolized sedation may reduce ventilator days, ICU length of stay, and certain safety events, providing external support for the observed direction of association [13]. In real-world practice, nurse-driven sedation protocols have been associated with favorable outcomes, including reduced ventilation duration [14]. In surgical ICU settings, nursing-driven protocols with explicit initiation criteria and titration rules have increased event-free ventilator time and reduced exposure to benzodiazepines and opioids, supporting the potential generalizability of this approach across ICU types [15].

Mechanistically, nurse-led targeted management may reduce deep-sedation exposure through a closed-loop workflow characterized by frequent assessment, timely titration, and rapid reassessment, thereby facilitating wakefulness and weaning progression while mitigating agitation-related risks [16,17]. The ABCDEF framework conceptualizes these elements as an integrated system linking assessment, sedation strategy, ventilator liberation, and early mobilization to reduce ICU-acquired complications and promote recovery [16]. However, implementation barriers (e.g., staffing, training, interprofessional coordination, workload, and local culture) can influence intervention intensity and sustainability, potentially explaining heterogeneity in delirium findings across settings [18].

Regarding delirium, we observed a lower delirium incidence in the nurse-led group but without statistical significance, which may reflect limited sample size, residual confounding, variation in implementation fidelity, and differential ascertainment due to more frequent delirium assessments in the nurse-led group. Meta-analytic evidence suggests that ABCDE/ABCDEF bundles are associated with improved delirium-related outcomes, yet heterogeneity is substantial, implying that larger samples and more rigorous monitoring of adherence may be required to detect consistent associations [19].

Several limitations should be emphasized. First, the retrospective observational design precludes causal inference, and the documentation-based classification of nurse-led management may be vulnerable to misclassification. Second, the sample size was small (n = 102), and several outcomes had few events, including delirium, 28-day mortality, reintubation within 48 hours, and unplanned extubation. To avoid model overfitting, adjusted analyses were intentionally parsimonious; therefore, important potential confounders were not fully controlled, including primary diagnosis, pre-exposure or pre-intervention ventilation time, respiratory severity (e.g., PaO2/FiO2 ratio and ventilator settings), sedative and opioid dose or cumulative sedative burden, neuromuscular blockade, de-escalation or weaning strategy, early mobilization, and adherence to the ABCDEF bundle. Third, process indicators such as assessment frequency and reassessment overlapped with the exposure definition and should be interpreted as implementation descriptors rather than independent outcomes. Fourth, the original retrospective screening log did not preserve total screened counts or reason-specific exclusion counts, limiting the completeness of the STROBE patient-flow report. These limitations may introduce residual confounding, information bias, and selection bias; prospective multicenter studies with standardized exposure definitions, comprehensive covariate capture, and formal competing-risk analyses are needed before causal effects can be inferred [20].

## 5. Conclusion

In mechanically ventilated adults in the AICU, nurse-led targeted sedation-analgesia management was associated with higher sedation target attainment, lower exposure to deep sedation and agitation, shorter duration of invasive mechanical ventilation, and shorter ICU length of stay. No significant differences were observed in key safety outcomes (unplanned extubation, reintubation within 48 hours, tracheostomy, or 28-day mortality), and delirium showed a nonsignificant downward trend. Because of the retrospective design, small sample size, limited adjustment, and residual confounding, these findings should be interpreted as hypothesis-generating associations rather than causal evidence of effectiveness.

## References

[1] Devlin JW, Skrobik Y, Gélinas C, Needham DM, Slooter AJC, Pandharipande PP, et al. Clinical practice guidelines for the prevention and management of pain, agitation/sedation, delirium, immobility, and sleep disruption in adult patients in the ICU. Crit Care Med. 2018;46(9):e825–73. doi: 10.1097/CCM.0000000000003299 30113379

[2] Sessler CN, Gosnell MS, Grap MJ, Brophy GM, O’Neal PV, Keane KA, et al. The Richmond Agitation-Sedation Scale: Validity and reliability in adult intensive care unit patients. Am J Respir Crit Care Med. 2002;166(10):1338–44. doi: 10.1164/rccm.2107138 12421743

[3] Gélinas C, Johnston C. Pain assessment in the critically ill ventilated adult: Validation of the Critical-Care Pain Observation Tool and physiologic indicators. Clin J Pain. 2007;23(6):497–505. doi: 10.1097/AJP.0b013e31806a23fb 17575489

[4] Keane KM. Validity and reliability of the critical care pain observation tool: A replication study. Pain Manag Nurs. 2013;14(4):e216–25. doi: 10.1016/j.pmn.2012.01.002 24315275

[5] Ely EW, Shintani A, Truman B, Speroff T, Gordon SM, Harrell FE, et al. Delirium as a predictor of mortality in mechanically ventilated patients in the intensive care unit. JAMA. 2004;291(14):1753–62. doi: 10.1001/jama.291.14.1753 15082703

[6] Kress JP, Pohlman AS, O’Connor MF, Hall JB. Daily interruption of sedative infusions in critically ill patients undergoing mechanical ventilation. N Engl J Med. 2000;342(20):1471–7. doi: 10.1056/NEJM200005183422002 10816184

[7] Girard TD, Kress JP, Fuchs BD, Thomason JWW, Schweickert WD, Pun BT, et al. Efficacy and safety of a paired sedation and ventilator weaning protocol for mechanically ventilated patients in intensive care (Awakening and Breathing Controlled trial): A randomised controlled trial. Lancet. 2008;371(9607):126–34. doi: 10.1016/S0140-6736(08)60105-1 18191684

[8] Pandharipande PP, Pun BT, Herr DL, Maze M, Girard TD, Miller RR, et al. Effect of sedation with dexmedetomidine vs lorazepam on acute brain dysfunction in mechanically ventilated patients: The MENDS randomized controlled trial. JAMA. 2007;298(22):2644–53. doi: 10.1001/jama.298.22.2644 18073360

[9] Barr J, Fraser GL, Puntillo K, Ely EW, Gélinas C, Dasta JF, et al. Clinical practice guidelines for the management of pain, agitation, and delirium in adult patients in the intensive care unit. Crit Care Med. 2013;41(1):263–306. doi: 10.1097/CCM.0b013e3182783b72 23269131

[10] Bugedo G, Tobar E, Aguirre M, Gonzalez H, Godoy J, Lira MT, et al. The implementation of an analgesia-based sedation protocol reduced deep sedation and proved to be safe and feasible in patients on mechanical ventilation. Rev Bras Ter Intensiva. 2013;25(3):188–96. doi: 10.5935/0103-507X.20130034 24213081 PMC4031854

[11] Pun BT, Balas MC, Barnes-Daly MA, Thompson JL, Aldrich JM, Barr J, et al. Caring for Critically Ill Patients with the ABCDEF Bundle: Results of the ICU Liberation Collaborative in Over 15,000 Adults. Crit Care Med. 2019;47(1):3–14. doi: 10.1097/CCM.0000000000003482 30339549 PMC6298815

[12] Minhas MA, Velasquez AG, Kaul A, Salinas PD, Celi LA. Effect of protocolized sedation on clinical outcomes in mechanically ventilated intensive care unit patients: A systematic review and meta-analysis of randomized controlled trials. Mayo Clin Proc. 2015;90(5):613–23. doi: 10.1016/j.mayocp.2015.02.016 25865475 PMC6469349

[13] Hernandez FLC, Rios MVS, Cardenas Bolivar YR, Alvarado Sanchez JI. Optimizing patient outcomes: A comprehensive evaluation of protocolized sedation in intensive care settings: a systematic review and meta-analysis. Eur J Med Res. 2024;29:255. doi: 10.1186/s40001-024-01839-y 38659054 PMC11044308

[14] Green S, Staffileno BA. Favorable outcomes after implementing a nurse-driven sedation protocol. Crit Care Nurse. 2021;41(6):29–35. doi: 10.4037/ccn2021625 34851385

[15] Kaplan JB, Eiferman DS, Porter K, MacDermott J, Brumbaugh J, Murphy CV. μImpact of a nursing-driven sedation protocol with criteria for infusion initiation in the surgical intensive care unit. J Crit Care. 2019;50:195–200. doi: 10.1016/j.jcrc.2018.11.029 30553990

[16] Marra A, Ely EW, Pandharipande PP, Patel MB. The ABCDEF Bundle in Critical Care. Crit Care Clin. 2017;33(2):225–43. doi: 10.1016/j.ccc.2016.12.005 28284292 PMC5351776

[17] Balas MC, Vasilevskis EE, Olsen KM, Schmid KK, Shostrom V, Cohen MZ, et al. Effectiveness and safety of the awakening and breathing coordination, delirium monitoring/management, and early exercise/mobility bundle. Crit Care Med. 2014;42(5):1024–36. doi: 10.1097/CCM.0000000000000129 24394627 PMC4105208

[18] Balas MC, Pun BT, Pasero C, Engel HJ, Perme C, Esbrook CL, et al. Common challenges to effective ABCDEF bundle implementation: The ICU liberation campaign experience. Crit Care Nurse. 2019;39(1):46–60. doi: 10.4037/ccn2019927 30710036

[19] Sosnowski K, Lin F, Chaboyer W, Ranse K, Heffernan A, Mitchell M. The effect of the ABCDE/ABCDEF bundle on delirium, functional outcomes, and quality of life in critically ill patients: A systematic review and meta-analysis. Int J Nurs Stud. 2023;138:104410. doi: 10.1016/j.ijnurstu.2022.104410 36577261

[20] Ranzani OT, Simpson ES, Augusto TB, Cappi SB, Noritomi DT, AMIL Critical Care Group. Evaluation of a minimal sedation protocol using ICU sedative consumption as a monitoring tool: A quality improvement multicenter project. Crit Care. 2014;18(5):580. doi: 10.1186/s13054-014-0580-3 25673553 PMC4234844

